# Systemic atopy and upper-airway disease define susceptibility to incident asthma after COVID-19 in Korea

**DOI:** 10.1038/s41467-026-74860-w

**Published:** 2026-06-27

**Authors:** You-Jung Choi, Young-Chan Kim, Sungho Bea, Ju-Young Shin, Hyesung Lee

**Affiliations:** 1 Department of Internal Medicine, Seoul National University College of Medicine, Seoul, Republic of Korea; 2 Biomedical Research Institute, Seoul National University Hospital, Seoul, Republic of Korea; 3 Division of Allergy and Clinical Immunology, Seoul National University Hospital, Seoul, Republic of Korea; 4 Division of Pharmacoepidemiology and Pharmacoeconomics, Brigham and Women’s Hospital, Boston, MA USA; 5 Department of Medicine, Harvard Medical School, Boston, MA USA; 6 School of Pharmacy, Sungkyunkwan University, Suwon, Republic of Korea; 7 Department of Medical Informatics, Kangwon National University College of Medicine, Chuncheon, Republic of Korea

**Keywords:** Asthma, Viral infection, Epidemiology

## Abstract

Incident asthma is an important respiratory sequela after COVID-19, but it is unclear which allergic phenotypes amplify risk. Using a linked nationwide Korean database of 3,987,182 individuals with confirmed severe acute respiratory syndrome coronavirus 2 infection, we compare claims-based incident asthma in those with pre-existing systemic atopy and/or upper-airway disease (allergic rhinitis, chronic rhinosinusitis, atopic dermatitis or food allergy) versus those without after 1:1 propensity score matching. During follow-up to 31 December 2022, participants with pre-existing disease have higher asthma incidence than matched controls (3.55 vs 2.13 per 1,000 person-years), with a hazard ratio of 1.66 (95% confidence interval 1.58–1.75). Asthma risk is elevated for each condition and increases with greater disease burden. These findings show that pre-existing allergic and upper-airway phenotypes stratify post-COVID incident asthma risk on a national scale, supporting targeted surveillance in high-risk subgroups.

## Abstract (web summary)

Authors conduct a cohort study using a nationwide Korean database. They show that people with allergic or upper-airway diseases before COVID-19 infection have higher subsequent asthma risk, which supports targeted follow-up in high-risk groups.

## Introduction

Individuals recovering from severe acute respiratory syndrome coronavirus 2 (SARS-CoV-2) infection frequently develop long-term respiratory diseases^1^. Specifically, asthma has emerged as a clinically relevant respiratory outcome following coronavirus disease 2019 (COVID-19), with several observational studies reporting a consistent increase in its risk^2–4^. Given its chronic course, recurrent exacerbations, and high healthcare burden, asthma is a major contributor to global respiratory morbidity, affecting over 260 million people worldwide^5^.

Mechanistically, SARS-CoV-2 infection may facilitate asthma development via suppression of epithelial type I/III interferons, activation of epithelial alarmins, including interleukin-33 (IL-33) and thymic stromal lymphopoietin (TSLP), and small-airway dysfunction with air-trapping and hyperinflation^6–9^. However, the extent to which these pathways are influenced by host immune profiles—particularly pre-existing systemic atopy and upper-airway diseases—remains poorly understood. Allergic diseases, such as allergic rhinitis, atopic dermatitis, and food allergy, share immune features with asthma and often progress as part of the atopic march. Chronic rhinosinusitis is heterogeneous, encompassing type 2 (T2)-high and non-T2 phenotypes; T2-high forms, often with nasal polyps, may involve immunoglobulin E (IgE) responses to staphylococcal enterotoxins^10,11^. Although multiple cohort studies have reported an increased incidence of post-COVID-19 asthma, most have focused on overall risk estimates. Whether susceptibility to incident asthma after SARS-CoV-2 infection is heterogeneous among survivors and patterned by pre-existing allergic or upper-airway inflammatory phenotypes has not been systematically evaluated. Distinguishing between these possibilities is important for understanding post-COVID asthma risk and for informing targeted post-COVID surveillance. Given these links to lower-airway disease, we assessed whether pre-existing systemic atopy (atopic dermatitis and food allergy) and upper-airway disease (allergic rhinitis and chronic rhinosinusitis), considered collectively and individually, predispose individuals with SARS-CoV-2 infection to develop incident asthma. We conducted a population-based cohort study using linked Korea Disease Control and Prevention Agency–National Health Insurance Service (KDCA–NHIS) data to quantify incident asthma risk after COVID-19 according to these pre-existing phenotypes.

## Results

### Baseline characteristics

**Fig. 1:**
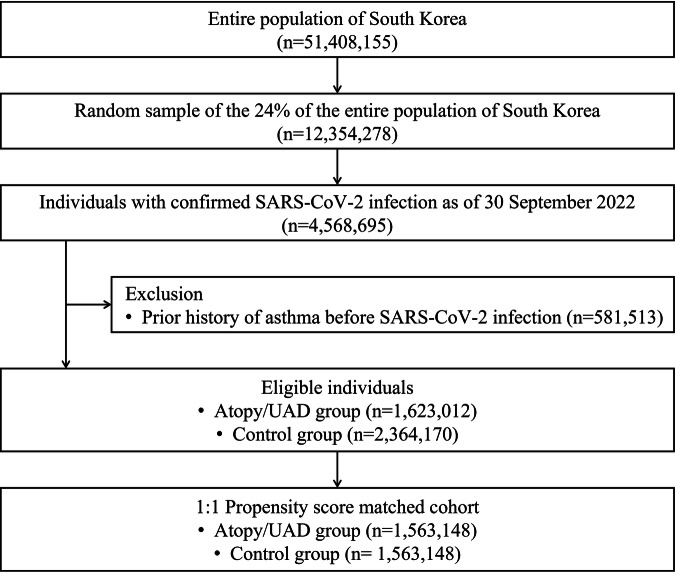
Study flow chart. Atopy/UAD, systemic atopy and/or upper-airway disease; SARS-CoV-2, severe acute respiratory syndrome coronavirus 2. Source data are provided as a Source data file.

Before propensity score matching (PSM), 1,623,012 individuals were included in the systemic atopy and/or upper-airway disease group (Atopy/UAD group), and 2,364,170 in the control group (Fig. 1). Individuals in the Atopy/UAD group were younger and included a higher proportion of males (Table 1). Differences were also observed in comorbidity profiles, including a lower prevalence of hypertension, dyslipidemia, and diabetes mellitus, but a higher prevalence of chronic obstructive pulmonary disease in the Atopy/UAD group. After 1:1 PSM, 1,563,148 individuals remained in each group, and all baseline characteristics were well-balanced across demographic variables, comorbidities, and co-medications (Table 1). Overall balance was also achieved across most baseline characteristics within each specific disease subgroup, including atopic dermatitis, food allergy, allergic rhinitis, and chronic rhinosinusitis (Supplementary Tables 3–6).

**Table 1:** Baseline characteristics before and after propensity score matching by pre-existing systemic atopy and upper-airway disease

Variables	Before propensity score matching			After propensity score matching		
	Atopy/UAD group	Control group	aSD	Atopy/UAD group	Control group	aSD
	(*n* = 1,623,012)	(*n* = 2,364,170)		(*n* = 1,563,148)	(*n* = 1,563,148)	
Mean age (SD), y	35.5 (21.1)	42.3 (20.3)	0.33	36.6 (20.8)	37.4 (20.0)	0.04
Sex, *n* (%)			0.08			0.07
Female	721,779 (44.5)	1,141,860 (48.3)		718,591 (46.0)	662,500 (42.4)	
Male	901,233 (55.5)	1,222,310 (51.7)		844,557 (54.0)	900,648 (57.6)	
Region of residence, *n* (%)			0.01			0.01
Urban	708,691 (43.7)	1,049,501 (44.4)		686,788 (43.9)	692,677 (44.3)	
Rural	914,321 (56.3)	1,314,669 (55.6)		876,360 (56.1)	870,471 (55.7)	
Income level, *n* (%)			0.02			0.00
Low (≤20%)	301,665 (18.6)	457,354 (19.4)		293,627 (18.8)	293,042 (18.8)	
Middle or high (>20%)	1,321,347 (81.4)	1,906,816 (80.7)		1,269,521 (81.2)	1,270,106 (81.3)	
Charlson comorbidity index, *n* (%)			0.09			0.05
0	1,317,981 (81.2)	1,919,934 (81.2)		1,267,752 (81.1)	1,272,919 (81.4)	
1–2	148,831 (9.2)	169,115 (7.2)		140,415 (9.0)	129,349 (8.3)	
>2	156,200 (9.6)	275,121 (11.6)		154,981 (9.9)	160,880 (10.3)	
Comorbidities						
Hypertension	194,179 (12.0)	367,147 (15.5)	0.10	193,690 (12.4)	198,981 (12.7)	0.01
Dyslipidemia	218,816 (13.5)	351,653 (14.9)	0.04	217,292 (13.9)	224,297 (14.4)	0.01
Diabetes mellitus	102,603 (6.3)	195,286 (8.3)	0.07	102,257 (6.5)	104,775 (6.7)	0.01
Chronic kidney disease	6842 (0.4)	13,644 (0.6)	0.02	6813 (0.4)	6427 (0.4)	0.00
COPD	32,097 (2.0)	30,419 (1.3)	0.05	27,248 (1.7)	26,455 (1.7)	0.00
Rheumatic disease	7736 (0.5)	11,026 (0.5)	0.00	7600 (0.5)	7326 (0.5)	0.00
Co-medications						
Immunomodulator	9120 (0.6)	10,892 (0.5)	0.01	8687 (0.6)	8108 (0.5)	0.01
Smoking			0.14			0.02
Never	580,550 (35.8)	908,790 (38.4)		579,346 (37.1)	572,642 (36.6)	
Ever	231,644 (14.3)	424,151 (17.9)		231,427 (14.8)	223,171 (14.3)	
Unknown	810,818 (50.0)	1,031,229 (43.6)		752,375 (48.1)	767,335 (49.1)	

aSDs were used to evaluate the balance of baseline characteristics between the two groups, with a threshold of 0.1 considered acceptable balance.

*aSD* absolute standardized difference, *SD* standard deviation, *COPD* chronic obstructive pulmonary disease, *Atopy/UAD* systemic atopy and/or upper-airway disease.

### Risk of claims-based incident asthma following COVID-19 infection

**Fig. 2:**
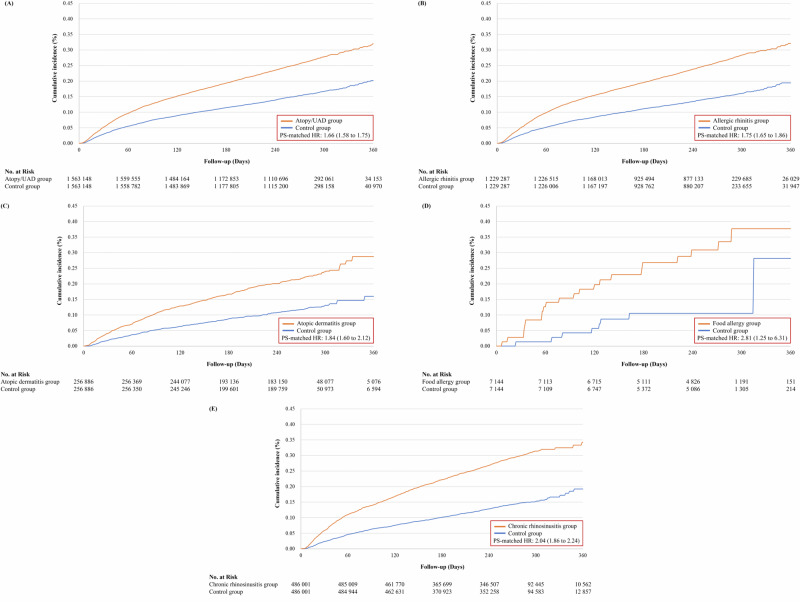
Cumulative incidence curves of claims-based incident asthma following COVID-19 infection after propensity score matching, according to pre-existing systemic atopy and/or upper-airway disease. **A** Composite exposure (systemic atopy and/or upper-airway disease). **B** Allergic rhinitis. **C** Atopic dermatitis. **D** Food allergy. **E** Chronic rhinosinusitis. Orange lines indicate the exposed group shown in each panel, and blue lines indicate the corresponding propensity score–matched control group. Cumulative incidence curves were plotted using the Kaplan-Meier method. Hazard ratios and 95% confidence intervals were estimated using Cox proportional hazards models and are shown within each panel. Atopy/UAD systemic atopy and/or upper-airway disease, CI confidence interval, COVID-19 coronavirus disease 2019, HR hazard ratio, PS propensity score. Source data are provided as a Source data file.

In the matched cohort, the incidence rate (IR) of claims-based incident asthma was significantly higher in the Atopy/UAD group (3.55 per 1000 person-years) than in the control group (2.13 per 1000 person-years), corresponding to a hazard ratio (HR) of 1.66 (95% confidence interval [CI], 1.58–1.75) (Table 2). Risks were higher across all specific pre-existing conditions, including atopic dermatitis (HR 1.84, 95% CI 1.60–2.12), allergic rhinitis (HR 1.75, 95% CI 1.65–1.86), food allergy (HR 2.81, 95% CI 1.25–6.31), and chronic rhinosinusitis (HR 2.04, 95% CI 1.86–2.24) (Table 2). The cumulative incidence curves for the two groups diverged early after the index date and continued to separate throughout the follow-up period (Fig. 2A); similar patterns were observed for each specific condition (Fig. 2B–E). Associations were consistent in sensitivity analyses using a stricter asthma definition and a minimum follow-up window (Supplementary Table 8).

**Table 2:** Risk of claims-based incident asthma following COVID-19 infection after propensity score matching, by pre-existing systemic atopy and/or upper-airway disease

Groups	No. of patients	No. of events	Sum of PYs	IR per 1000 PYs (95% CI)	HR (95% CI)
Systemic atopy and/or upper-airway disease					
Control group	1,563,148	2300	1,079,289	2.13 (2.05–2.22)	1.00 (reference)
Atopy/UAD group	1,563,148	3808	1,073,329	3.55 (3.44–3.66)	1.66 (1.58–1.75)
Specific disease					
Allergic rhinitis					
Without allergic rhinitis	1,229,287	1745	849,609	2.05 (1.96–2.15)	1.00 (reference)
With allergic rhinitis	1,229,287	3044	845,037	3.60 (3.48–3.73)	1.75 (1.65–1.86)
Atopic dermatitis					
Without atopic dermatitis	256,886	297	180,038	1.65 (1.47–1.85)	1.00 (reference)
With atopic dermatitis	256,886	537	176,350	3.05 (2.80–3.31)	1.84 (1.60–2.12)
Food allergy					
Without food allergy	7144	8	4919	1.63 (0.81–3.25)	1.00 (reference)
With food allergy	7144	22	4774	4.61 (3.03–7.00)	2.81 (1.25–6.31)
Chronic rhinosinusitis					
Without chronic rhinosinusitis	486,001	658	337,916	1.95 (1.80–2.10)	1.00 (reference)
With chronic rhinosinusitis	486,001	1333	334,467	3.99 (3.78–4.21)	2.04 (1.86–2.24)

For each specific condition, analyses were conducted in separate 1:1 propensity score-matched cohorts comparing individuals with the condition to matched controls without the condition. IRs were calculated using Poisson regression models, and HRs were estimated using Cox proportional hazards models.

*Atopy/UAD* systemic atopy and/or upper-airway disease, *CI* confidence interval, COVID-19 coronavirus disease 2019, *HR* hazard ratio, *IR* incidence rate, *PY* person-years.

### Subgroup and sensitivity analyses

**Fig. 3:**
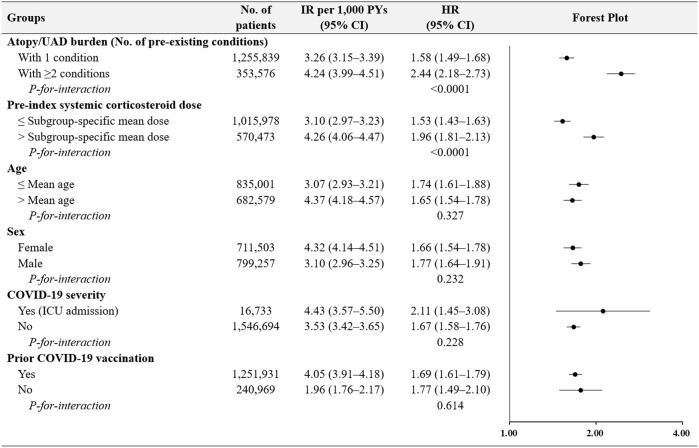
Subgroup analyses of the risk of claims-based incident asthma following COVID-19 infection after propensity score matching, by pre-existing systemic atopy and/or upper-airway disease. Points and horizontal lines indicate hazard ratios (HRs) and 95% confidence intervals (CIs) from Cox proportional hazards models comparing the Atopy/UAD group with matched controls within each stratum. P-for-interaction values were calculated using two-sided Wald tests for group-by-stratum interaction terms in Cox proportional hazards models; no adjustment was made for multiple comparisons. *P* values < 0.0001 are reported as <0.0001. Atopy/UAD systemic atopy and/or upper-airway disease, CI confidence interval, COVID-19 coronavirus disease 2019, HR hazard ratio, ICU intensive care unit, IR incidence rate, PY person-years. Source data are provided as a Source data file.

Subgroup analyses showed evidence of effect modification by the disease burden (Fig. 3 and Supplementary Table 7). Individuals with one allergic or upper-airway condition had an IR of 3.26 per 1000 person-years compared with matched controls (IR 2.06), corresponding to a hazard ratio (HR) of 1.58 (95% CI 1.49–1.68). Those with two or more conditions had an IR of 4.24 per 1000 person-years versus matched controls (IR 1.73), with an HR of 2.44 (95% CI 2.18–2.73). The monotonic increase and significant interaction (P-for-interaction <0.0001) indicated a graded association between pre-existing disease burden and post-COVID asthma risk.

A similar pattern was observed for systemic corticosteroid exposure. Participants receiving doses above the subgroup-specific mean had an IR of 4.26 per 1000 person-years compared with 3.10 among those at or below the subgroup-specific mean. Compared with matched controls within each stratum, the HR was 1.53 (95% CI 1.43–1.63) among participants at or below the mean dose and 1.96 (95% CI 1.81–2.13) among those above the mean (P-for-interaction <0.0001).

In contrast, no significant effect modification was observed by age, sex, COVID-19 severity, or vaccination status.

Results from all sensitivity analyses were generally consistent with those of the main findings (Supplementary Table 8), across analyses addressing residual confounding by smoking status, outcome misclassification, and duration of follow-up. In calendar-time-stratified analyses comparing infections confirmed before and after 1 March 2022, the association remained consistent with the main findings (Supplementary Table 9). The propensity score-matched hazard ratio was 1.52 (95% CI, 1.39–1.67) before March 2022 and 1.72 (95% CI, 1.61–1.83) thereafter. In lag-time sensitivity analyses starting follow-up at 3 and 6 months after the index date, the associations remained generally consistent with the primary findings (Supplementary Table 10), making it less likely that the observed association is explained solely by transient early post-infectious respiratory symptoms. In sensitivity analyses applying extended washout periods of 2 and 3 years before the index date, the association remained consistent with the primary findings (Supplementary Table 11), with hazard ratios (HRs) of 1.64 (95% CI, 1.56–1.73) and 1.66 (95% CI, 1.58–1.75), respectively. After propensity score re-matching incorporating pre-index healthcare utilization, the association was attenuated but remained elevated (HR 1.37, 95% CI 1.30–1.45; Supplementary Table 12).

## Discussion

In this nationwide propensity-matched cohort of COVID-19 survivors, pre-existing systemic atopy (atopic dermatitis and food allergy) and upper-airway diseases (allergic rhinitis and chronic rhinosinusitis) were each associated with an increased risk of claims-based incident asthma. Associations were observed across individual conditions. In subgroup analyses, asthma risk increased with greater severity of pre-existing diseases, reflected by a higher number of systemic atopy or upper-airway conditions and greater pre-index systemic corticosteroid exposure. Sensitivity analyses produced consistent results across alternative definitions and populations.

Collectively, these results indicate that the risk of claims-based incident asthma after SARS-CoV-2 infection varies systematically by pre-existing systemic atopy and upper-airway inflammatory phenotypes. Rather than viewing post-COVID asthma solely as a direct consequence of viral injury, our findings are consistent with a host-susceptibility framework in which underlying allergic and upper-airway disease may amplify vulnerability to post-viral lower-airway disease and thereby help identify higher-risk subgroups for targeted post-COVID surveillance. Because this infected-only, claims-based design cannot directly quantify interaction relative to uninfected populations or establish a biological mechanism, these observations should be interpreted as biologically plausible risk stratification rather than mechanistic proof. The elevated risks across allergic phenotypes are biologically plausible and align with both T2 and non-T2 pathways^12–17^, although our data lacked biomarkers to confirm endotypes. The higher HRs for allergic rhinitis (HR 1.75) and for the claims-based chronic rhinosinusitis phenotype (HR 2.04) support the united airway framework, whereby pre-existing upper-airway inflammation may mark susceptibility to lower-airway pathology^18,19^. Within this broader upper-airway phenotype, both T2-prone and non-T2 mechanisms may be relevant. In T2-high profiles—more typical of chronic rhinosinusitis with nasal polyps—epithelial alarmins, such as IL-33 and TSLP, may amplify downstream inflammation and promote post-viral airway remodeling, and IgE responses (e.g., to staphylococcal enterotoxins) are frequently implicated^10,11,15,16^. In parallel, non-T2 axes are relevant: Th17-dominant/neutrophilic inflammation and neutrophil extracellular traps (NETs), demonstrated in severe COVID-19 lung tissue, may sustain epithelial injury independent of classic T2 signals^13,14,17^. Although our claims-based analysis could not stratify chronic rhinosinusitis by endotype or polyp status, these distinct pathways underscore its heterogeneity and the value of future biomarker-integrated studies^20^.

The higher risk observed with atopic dermatitis is consistent with an adult analog of the atopic march^21^. Although the estimate for food allergy was less precise because of the small number of events, its direction was broadly aligned with the same systemic atopy pattern. However, given the wide confidence interval, the food allergy finding should be interpreted cautiously as hypothesis-generating and requires confirmation in larger cohorts. Mechanistically, the underlying T2-skewed mucosa in these conditions may blunt early antiviral control—for instance, IgE-mediated signaling on plasmacytoid dendritic cells can suppress type I interferon production, while IL-4/IL-13 reinforce epithelial-barrier dysfunction—thereby favoring airway hyperresponsiveness and remodeling in the post-viral window^12,16,22^. This pro-asthmatic milieu may be further amplified by COVID-19-related structural and neurogenic sequelae, including persistent small-airways disease on expiratory chest computed tomography and cough-reflex hypersensitivity, which can unmask or accelerate incident asthma symptoms in susceptible adults^6,23^. Accordingly, our findings are hypothesis-generating for virus-dependent risk amplification, potentially involving convergent virus–epithelium–alarmin pathways that extend beyond SARS-CoV-2^15,16^. These observations suggest that pre-existing allergic and upper-airway phenotypes may increase susceptibility to post-viral lower-airway disease through host–virus interactions.

Risks were higher with multimorbidity and higher pre-index systemic corticosteroid exposure. The step-up in hazard from one condition to ≥2 conditions supports a graded association by condition count in post-COVID asthma, consistent with a cumulative-burden effect. The stronger association among those with higher pre-index systemic corticosteroid exposure most likely reflects confounding by indication: individuals with more active or severe allergic disease are both more likely to receive systemic corticosteroids and more likely to have greater underlying disease activity that predisposes to post-viral asthma. Importantly, pre-index systemic corticosteroid exposure was defined using indication-specific prescriptions—systemic corticosteroids dispensed in claims carrying the corresponding Atopy/UAD diagnosis codes—to minimize capture of corticosteroids prescribed for unrelated conditions. This supports its interpretation as a proxy for disease activity and severity. While prior studies have suggested that systemic corticosteroids may be associated with delayed viral clearance^24,25^, our observational data cannot disentangle pharmacologic effects from disease severity and therefore do not support causal inference regarding a direct pharmacologic pathway.

Clinically, these results suggest risk-stratified post-COVID follow-up for patients with allergic diseases and/or upper-airway disease, especially those with multimorbidity or higher pre-index steroid exposure. Persistent respiratory symptoms may warrant spirometry and, where available, fractional exhaled nitric oxide testing for early detection. An integrated airways approach, including active management of rhinitis and sinusitis, may help reduce lower-airway risk. Future research should evaluate whether T2-targeted biologics used in selected upper-airway endotypes (e.g., dupilumab for chronic rhinosinusitis with nasal polyps) alter post-COVID asthma risk in multimorbid patients^26^, and if biomarker-defined endotypes improve prediction.

The strengths of this study include its nationwide scope, enhancing generalizability, the use of propensity score matching to balance multiple covariates, and sensitivity analyses supporting the consistency of the findings. Nevertheless, several limitations warrant consideration. First, the study relied on claims-based definitions without spirometry or biomarkers and therefore cannot distinguish between asthma endotypes (e.g., T2-high versus non-T2) or confirm variable airflow limitation objectively. Our central contribution is not endotype assignment but population-level susceptibility stratification. Future studies integrating spirometry, biomarkers, and detailed clinical phenotyping will be necessary to clarify the underlying endotypes and mechanisms.

Second, SARS-CoV-2 infection was identified through confirmed cases recorded in the national registry. During the study period, confirmation was primarily based on laboratory polymerase chain reaction (PCR) testing, although national testing policies evolved over time; from mid-March 2022, positive rapid antigen results were also accepted as confirmed cases in certain settings. Individuals tested solely by home-based antigen kits without formal confirmation would not have been captured. The association remained consistent across calendar periods, suggesting that testing changes are unlikely to explain the findings, although under-ascertainment of milder infections may limit generalizability.

Third, differential healthcare utilization may have introduced detection bias, as individuals with pre-existing Atopy/UAD may have greater baseline contact with the healthcare system. To address this concern, we re-matched on propensity scores incorporating pre-index all-cause and respiratory-related outpatient visits and hospitalizations; the association was attenuated but remained elevated, suggesting that differences in baseline healthcare contact do not fully explain the observed association. Nevertheless, residual confounding by unmeasured factors (e.g., environmental exposures, SARS-CoV-2 variants, or genetic susceptibility) cannot be excluded.

Fourth, because claims history was available from 2019 onward, some individuals with historically prevalent but clinically inactive asthma may have been misclassified as incident cases. Fifth, the follow-up period (2020–2022) may be insufficient to capture longer-term respiratory sequelae, and some post-COVID respiratory conditions could mimic asthma in administrative data. Additional limitations include limited precision for food allergy due to small numbers and restricted generalizability beyond the Korean population. As the cohort comprised only individuals with confirmed SARS-CoV-2 infection, the findings reflect risk amplification among infected individuals and do not quantify interactions relative to uninfected populations. Finally, heterogeneity within chronic rhinosinusitis (e.g., polyp status or inflammatory endotypes) could not be assessed, and extrapolation to non-SARS-CoV-2 respiratory infections remains speculative.

In conclusion, pre-existing systemic atopy and upper-airway diseases were associated with an increased risk of claims-based incident asthma following COVID-19 infection. The association was consistent across individual conditions and was amplified in patients with greater disease burden or higher systemic corticosteroid use. These findings suggest that host allergic profiles may influence adverse respiratory outcomes after viral infection and underscore the need for tailored post-COVID surveillance in patients with allergic or upper-airway diseases. Further studies integrating immunologic and clinical data are warranted to identify high-risk endotypes and guide targeted preventive interventions.

## Methods

### Ethical approval and reporting considerations

This study complied with all relevant ethical regulations. The study protocol was approved by the Institutional Review Board of Sungkyunkwan University (SKKU 2023-04-037), and the requirement for informed consent was waived because the study used de-identified administrative data. Access to the KDCA COVID-19 NHIS Cohort (K-COV-N) database was approved under application number NHIS-2023-1-508. Participant compensation was not applicable because this was a retrospective database study.

### Study design and data source

We conducted a retrospective cohort study using the KDCA COVID-19 NHIS Cohort (K-COV-N) database from 2019 to 2022^27^. This database was established to facilitate research on COVID-19 in South Korea by linking registry data for SARS-CoV-2 infection and COVID-19 vaccination from the KDCA, claims and health screening program database from the NHIS, and mortality database from Statistics Korea. Under South Korea’s mandatory universal health coverage through a single-payer system, the NHIS data cover approximately 98% of the national population. The K-COV-N cohort was constructed via stratified random sampling, comprising 12,354,278 individuals (24% of the total Korean population of 51,408,155 in 2022). First, the SARS-CoV-2 infection database included all individuals with confirmed COVID-19 recorded in the KDCA-linked database. During the study period, confirmation was primarily based on laboratory PCR testing, although national testing policies evolved over time. The database contained information on the date of confirmation, reporting institution, route of infection, and cause of infection. Second, the COVID-19 vaccination database included the date of vaccination, vaccine type, and number of doses. Third, the claims database comprised comprehensive healthcare utilization information, including diagnostic codes based on the International Classification of Diseases, 10th Revision (ICD-10), prescription records detailing drug name, dosage, administration route, prescribing date, and duration of supply, as well as procedure data from inpatient, outpatient, and emergency department encounters. Fourth, the health screening program database contained results from laboratory tests and self-reported lifestyle questionnaires, collected biennially through a government-funded national medical screening service. Finally, the mortality database included the date of death and cause of death, enabling complete follow-up for death information.

The NHIS data include administratively recorded sex, recorded as male or female, which was used as a baseline covariate and subgroup variable. Gender identity information was not available in the administrative database. Age and sex distributions are reported in Table 1 and Supplementary Tables 3–6.

#### Study population and exposure

We included all individuals with confirmed SARS-CoV-2 infection up to 30 September 2022 to ensure a minimum follow-up of three months. The index date was defined as the first date of SARS-CoV-2 infection recorded in the registry. To establish an incident cohort, individuals with any record of asthma diagnosis before the index date were excluded using all available claims history in K-COV-N prior to the index date (from the start of available records in 2019 to the day before the index date).

The primary exposure was defined as a composite of pre-existing systemic atopy and upper-airway diseases, including allergic rhinitis, atopic dermatitis, food allergy, and chronic rhinosinusitis, before the index date. Individuals with any of these pre-existing conditions were classified as the Atopy/UAD group, and those without as the control group. All pre-existing diseases were identified based on ICD-10 diagnostic codes combined with prescription records for disease-specific medications, except for food allergy, which was identified using ICD-10 diagnostic codes alone. Detailed information on diagnostic codes and disease-specific medications is presented in Supplementary Tables 1 and 2. Secondary exposures were defined as the individual component diseases assessed separately.

### Outcome and follow-up

The study outcome was claims-based incident asthma, defined according to a prespecified algorithm requiring at least one record of diagnostic codes (ICD-10 codes J45 or J46) and a prescription for asthma-related medications during follow-up^28^. Asthma-related medications were defined as including inhaled or systemic corticosteroids, bronchodilators, leukotriene receptor antagonists, and xanthine derivatives (Supplementary Table 2). All individuals were followed from the index date until the earliest occurrence of the outcome of interest, death, or the end of the study period (31 December 2022).

### Potential confounders

We considered a wide range of potential confounders measured at the index date or during the year preceding the index date, including demographics (age, sex, region of residence, and income level), comorbidities (hypertension, dyslipidemia, diabetes, chronic kidney disease, chronic obstructive pulmonary disease, and rheumatic disease), use of immunomodulators, Charlson comorbidity index, and smoking status. Use of immunomodulators served as an indicator of immunomodulatory status and was identified from prescription records of cyclosporine, tacrolimus, azathioprine, mycophenolate mofetil, sirolimus, everolimus, and alemtuzumab^29^. Smoking status was available for a subset of participants from the national health screening database and was evaluated in sensitivity analyses.

### Statistical analysis

Baseline characteristics were summarized as means with standard deviations (SDs) for continuous variables and as numbers with percentages for categorical variables. To account for confounding, we conducted 1:1 PSM between the Atopy/UAD and control groups using a greedy nearest-neighbor matching algorithm without replacement. Propensity score, or the probability of having pre-existing systemic atopy and upper-airway diseases, was estimated using a logistic regression model based on all baseline characteristics except for smoking status because of a high proportion of missing values. The absolute standardized difference was used to evaluate the balance of baseline characteristics between the two groups, with a threshold of 0.1 considered acceptable balance. We calculated incidence rates per 1000 person-years with 95% confidence intervals (CIs) using a Poisson regression model, estimated HRs with 95% CIs using a Cox proportional hazards model, and plotted cumulative incidence curves to illustrate the divergence in outcome occurrence between the two groups during follow-up. A two-sided *P* value of <0.05 was considered statistically significant. All data management and analyses were performed using SAS Enterprise Guide, version 7.1 (SAS Institute, Cary, NC, USA).

### Subgroup and sensitivity analyses

We conducted several subgroup analyses to evaluate potential effect modifications according to the severity of pre-existing diseases, COVID-19–related factors, and baseline characteristics. The severity of pre-existing diseases was represented by the mean pre-index systemic corticosteroid dose (≤mean vs. >mean) and the number of pre-existing diseases (1 vs. ≥2) before the index date.

For subgroup analyses, pre-index systemic corticosteroid exposure was operationalized as the mean dispensed systemic corticosteroid dose per prescription. Systemic corticosteroids were identified using the anatomical therapeutic chemical code H02AB (glucocorticoids; systemic; Supplementary Table 2). For each disease-specific subgroup (allergic rhinitis, chronic rhinosinusitis, atopic dermatitis, and food allergy), we identified all systemic corticosteroid prescriptions from the start of available records in the K-COV-N database to the day before the index date that were issued in claims carrying the corresponding disease diagnosis code (i.e., indication-specific systemic corticosteroids; Supplementary Table 1). We summed the total dispensed systemic corticosteroid dose across eligible prescriptions and divided by the number of prescriptions to derive an individual-level mean dose. This measure was calculated separately within each disease-specific subgroup, and participants were dichotomized at the subgroup-specific mean (≤mean vs. >mean). Participants without eligible pre-index systemic corticosteroid prescriptions were assigned a mean dose of 0.

COVID-19-related factors included admission to the intensive care unit (yes vs. no) at the index date and prior COVID-19 vaccination (yes vs. no) before the index date. The study population was further stratified by age (≤mean vs. >mean) and sex (female vs. male).

We also conducted several sensitivity analyses to assess the consistency of our findings. First, to account for potential residual confounding due to missing smoking data, we restricted the study population to participants with available smoking status data. We then performed an additional analysis stratified by smoking status (never vs. ever smokers). Second, to account for potential outcome misclassification, we redefined the outcome using a stricter claims-based incident asthma definition requiring at least two records of asthma diagnosis with asthma-related prescription. Third, to ensure sufficient follow-up for identifying claims-based incident asthma, we restricted the analysis to participants who were followed up for at least 6 months. Fourth, to address potential under-ascertainment of SARS-CoV-2 infection due to home-based antigen testing without subsequent PCR confirmation, we performed calendar-time-stratified analyses comparing infections confirmed before and after 1 March 2022. This cutoff was chosen to reflect the transition period during the omicron wave in Korea, when rapid antigen testing became more widely used, and positive rapid antigen results were accepted as confirmed cases in certain settings^30,31^. Fifth, to reduce the possibility that transient post-infectious respiratory symptoms or short-term bronchial hyperresponsiveness were misclassified as incident asthma, we conducted lag-time sensitivity analyses excluding outcomes occurring within 3 and 6 months after the index date. In these analyses, individuals were followed from the end of each lag period, and events occurring during the lag period were excluded (Supplementary Table 10). Sixth, to minimize potential misclassification of prevalent asthma due to the limited availability of claims history prior to 2019, we performed sensitivity analyses applying extended washout periods of 2 and 3 years before the index date (Supplementary Table 11). Finally, to evaluate the potential for detection bias arising from differences in baseline healthcare utilization, we re-estimated propensity scores with additional inclusion of healthcare utilization measures during the 1 year prior to the index date and repeated 1:1 nearest-neighbor matching. These measures included all-cause outpatient visits (continuous), all-cause hospitalizations (categorical, 0 vs. ≥1), respiratory-related outpatient visits (continuous), and respiratory-related hospitalizations (categorical, 0 vs. ≥1). Respiratory-related healthcare utilization was defined using ICD-10 codes J00–J99, excluding J45–J46 (Supplementary Table 12).

### Reporting summary

Further information on research design is available in the Nature Portfolio Reporting Summary linked to this article.

## Supplementary information

Supplementary Information

Peer Review File

Reporting Summary

## Source data

Source Data

## Data Availability

Individual-level data used in this study were derived from Korean national claims data and infectious disease registry data. These data cannot be deposited in a public repository or shared publicly by the authors because they contain sensitive personal health information and are subject to legal restrictions and data-use agreements imposed by the data custodians. The authors are not permitted to distribute, transfer, or provide access to the individual-level data to third parties. Researchers seeking access to National Health Insurance Service data may submit data access requests through the National Health Insurance Sharing Service website: https://nhiss.nhis.or.kr/. Access to the linked KDCA COVID-19 NHIS Cohort (K-COV-N) data is subject to review and approval by the relevant data custodians, including the National Health Insurance Service and the relevant infectious disease registry data custodian, through their formal data application procedures. Data access is subject to execution of data-use agreements and compliance with applicable ethical and legal requirements. Access is restricted to approved researchers for approved research purposes and may be provided only within the approved data environment specified by the data custodians. The expected timeframe for response to access requests and the duration of data availability after approval are determined by the respective data custodians according to their data access policies. Source data are provided with this paper.

## Code availability

The analytic code used in this study is publicly available on GitHub at https://github.com/isley88/NCOMMS-25-105057A and has been archived in Zenodo under 10.5281/zenodo.20373495. Owing to legal restrictions on the underlying individual-level Korean national claims and infectious disease registry data, the code cannot be fully executed without authorized data access^32^.
