# Supplementary material for: Systemic atopy and upper-airway disease define susceptibility to incident asthma after COVID-19 in Korea
Source: Nat Commun. 2026 Jun 27;17:8042. doi: 10.1038/s41467-026-74860-w (PMC13454191; doi:10.1038/s41467-026-74860-w)
Supplement: Supplementary file 1 [file 41467_2026_74860_MOESM1_ESM.pdf]

Systemic atopy and upper-airway disease define susceptibility to incident asthma after COVID-19 in Korea

You-Jung Choi, Young-Chan Kim, Sungho Bea, Ju-Young Shin, Hyesung Lee

Supplementary Information

Supplementary Table 1. Definition of variables

Supplementary Table 2. Definition of medications with ATC codes

Supplementary Table 3. Baseline characteristics of the study population by the history of allergic rhinitis

Supplementary Table 4. Baseline characteristics of the study population by the history of atopic dermatitis

Supplementary Table 5. Baseline characteristics of the study population by the history of food allergy

Supplementary Table 6. Baseline characteristics of the study population by the history of chronic rhinosinusitis

Supplementary Table 7. Subgroup analyses of the risk of claims-based incident asthma following COVID-19 infection after propensity score matching, by pre-existing systemic atopy and upper-airway disease

Supplementary Table 8. Sensitivity analyses of the risk of claims-based incident asthma following COVID-19 infection after propensity score matching, by pre-existing systemic atopy and upper-airway disease

Supplementary Table 9. Sensitivity analyses of the risk of claims-based incident asthma following COVID-19 infection after propensity score matching, by pre-existing systemic atopy and upper-airway disease: Calendar-time stratified analyses (before vs after March

2022)

Supplementary Table 10. Sensitivity analyses of the risk of claims-based incident asthma following COVID-19 infection after propensity score matching, by pre-existing systemic atopy and upper-airway disease: Lag-time analyses excluding early outcomes

Supplementary Table 11. Sensitivity analyses of the risk of claims-based incident asthma following COVID-19 infection after propensity score matching, by pre-existing systemic atopy and upper-airway disease: Analyses applying 2- and 3-year washout periods

Supplementary Table 12. Sensitivity analyses of the risk of claims-based incident asthma following COVID-19 infection after propensity score matching, by pre-existing systemic atopy and upper-airway disease: Analyses with propensity score re-estimation incorporating healthcare utilization

Supplementary Table 1. Definition of variables

Covariates	ICD-10 codes	Comments
**Study population**		
Allergic rhinitis	J30.1, J30.2, J30.3, J30.4, J30.5, J30.8, J30.9	≥ 1 record during hospitalization or outpatient clinic with a minimum of 1 prescription of any of the following: antihistamines, nasal steroids, systemic corticosteroids, or leukotriene antagonists
Atopic dermatitis	L20	≥ 1 record during hospitalization or outpatient clinic with a minimum of 1 prescription of any of the following: topical steroids, systemic corticosteroids, topical calcineurin inhibitor, azathioprine, cyclosporine, mycophenolate mofetil, methotrexate, dupilumab, or systemic JAK inhibitor
Chronic rhinosinusitis	J31.0, J32, J32.0, J32.1, J32.2, J32.3, J32.4, J32.8, J32.9	≥ 1 record during hospitalization or outpatient clinic with a minimum of 1 prescription of any of the following: antihistamines, nasal steroids, systemic corticosteroids, leukotriene antagonists, or macrolide antibiotics
Food allergy	K52.2, L27.2, L23.6, T78.0, T78.1<br>Z91.01 Food allergy status<br>Z91.010 Allergy to peanuts<br>Z91.011 Allergy to milk products<br>Z91.012 Allergy to eggs<br>Z91.013 Allergy to seafood	≥ 1 record during hospitalization or outpatient clinic

	Z91.018 Allergy to other foods<br>Z91.02 Food additives allergy status	
**Outcome**		
Asthma	J45-46	≥ 1 record during hospitalization or outpatient clinic with a minimum of 1 prescription of asthma-related medication, including inhaled or systemic corticosteroids, bronchodilators, leukotriene receptor antagonists, or xanthine derivatives
**Comorbidities**		
Hypertension	I10-I13, I15	≥ 1 record during hospitalization or outpatient clinic
Diabetes mellitus	E11-E14	≥ 1 record during hospitalization or outpatient clinic
Dyslipidemia	E78	≥ 1 record during hospitalization or outpatient clinic
Rheumatic disease	Rheumatoid arthritis (M05), Sjögren's syndrome (M35), systemic lupus erythematosus (M32), and Behcet's disease (M35.2)	≥ 1 record during hospitalization or outpatient clinic
Chronic kidney disease	N18, N19	≥ 1 record during hospitalization or outpatient clinic
Chronic obstructive lung disease	J41, J42, J43, J44	≥ 1 record during hospitalization or outpatient clinic

Abbreviations: ICD-10, International Classification of Diseases, 10th Revision.

Supplementary Table 2. Definition of medications with ATC codes

Category	ATC Codes (Description)
Antihistamines (systemic)	R06A (antihistamines for systemic use)
Nasal steroids (intranasal)	R01AD (nasal corticosteroids)
Systemic corticosteroids	H02AB (glucocorticoids; systemic)
Leukotriene receptor antagonists (LTRA)	R03DC (leukotriene receptor antagonists)
Topical steroids	D07 (dermatological corticosteroids; overall)
Topical calcineurin inhibitor	D11AH (e.g., tacrolimus/pimecrolimus)
Azathioprine	L04AX01
Cyclosporine	L04AD01
Mycophenolate mofetil	L04AA06
Methotrexate	L04AX03 (immunosuppressive)
Dupilumab	D11AH05
JAK inhibitor - Systemic (primarily immunosuppressive)	L04AF; D11AH08
Macrolide antibiotics	J01FA
Inhaled corticosteroid (ICS)	R03BA, R03AK, R03AL(08,09,11,12) (ICS alone and ICS/LABA (+/- LAMA))
Bronchodilators	R03AC (β2-agonists, inhalants), R03BB (anticholinergics, inhalants) ± R03CC (β2-agonists, systemic)
Xanthine derivatives	R03DA
Anti-hypertensives – thiazide	C03A
Anti-hypertensives – loop diuretics	C03C
Anti-hypertensives – aldosterone antagonist	C03DA
Anti-hypertensives – alpha-blocker	C02CA
Anti-hypertensives – beta-blocker	C07 (specify C07A for plain if needed)
Anti-hypertensives – calcium-channel blocker	C08
Angiotensin-converting-enzyme (ACE) inhibitor	C09A (plain) / C09B (combinations)

Angiotensin II receptor blocker (ARB)	C09C (plain) / C09D (combinations)
Anti-diabetic – metformin	A10BA (biguanides)
Anti-diabetic – sulfonylureas	A10BB
Anti-diabetic – meglitinides	A10BX (other blood glucose-lowering drugs; includes repaglinide/nateglinide)
Anti-diabetic – thiazolidinediones	A10BG
Anti-diabetic – Dipeptidyl peptidase 4 inhibitors	A10BH
Anti-diabetic – α-glucosidase inhibitors	A10BF
Anti-diabetic – insulin	A10A
Lipid-lowering agents	C10 (lipid modifying agents; e.g., statins C10AA)

Abbreviations: ATC, Anatomical Therapeutic Chemical code.

Supplementary Table 3. Baseline characteristics of the study population by the history of allergic rhinitis

Variables	Before propensity score matching		aSD	After propensity score matching		aSD
	With allergic rhinitis (n=1,253,260)	Without allergic rhinitis (n=2,364,170)		With allergic rhinitis (n=1,229,287)	Without allergic rhinitis (n=1,229,287)	
Mean age (SD), y	35.6 (21.2)	42.3 (20.3)	0.32	36.1 (21.0)	36.6 (20.6)	0.02
Sex, n (%)			0.08			0.06
Female	554,514 (44.3)	1,141,860 (48.3)		553,568 (45.0)	519,948 (42.3)	
Male	698,746 (55.8)	1,222,310 (51.7)		675,719 (55.0)	709,339 (57.7)	
Region of residence, n (%)			0.03			0.00
Urban	536,168 (42.8)	1,049,501 (44.4)		529,252 (43.1)	529,524 (43.1)	
Rural	717,092 (57.2)	1,314,669 (55.6)		700,035 (57.0)	699,763 (56.9)	
Income level, n (%)			0.02			0.00
Low (≤20%)	232,884 (18.6)	457,354 (19.4)		230,955 (18.8)	231,203 (18.8)	
Middle or High (>20%)	1,020,376 (81.4)	1,906,816 (80.7)		998,332 (81.2)	998,084 (81.2)	
Charlson Comorbidity Index, n (%)			0.09			0.00
0	1,013,904 (80.9)	1,919,934 (81.2)		993,727 (80.8)	993,757 (80.8)	
1-2	117,801 (9.4)	169,115 (7.2)		114,307 (9.3)	109,745 (8.9)	
>2	121,555 (9.7)	275,121 (11.6)		121,253 (9.9)	125,785 (10.2)	
Comorbidities						
Hypertension	151,574 (12.1)	367,147 (15.5)	0.10	151,423 (12.3)	154,173 (12.5)	0.01
Dyslipidemia	169,949 (13.6)	351,653 (14.9)	0.04	169,592 (13.8)	174,323 (14.2)	0.01
Diabetes mellitus	79,665 (6.4)	195,286 (8.3)	0.07	79,572 (6.5)	81,181 (6.6)	0.01
Chronic kidney disease	5,237 (0.4)	13,644 (0.6)	0.02	5,232 (0.4)	4,507 (0.4)	0.01
COPD	25,155 (2.0)	30,419 (1.3)	0.06	22,893 (1.9)	23,742 (1.9)	0.01
Rheumatic disease	5,848 (0.5)	11,026 (0.5)	0.00	5,824 (0.5)	5,274 (0.4)	0.01
Co-medications						
Immunomodulator	5,944 (0.5)	10,892 (0.5)	0.00	5,907 (0.5)	5,275 (0.4)	0.01
Smoking			0.14			0.05
Never	449,908 (35.9)	908,790 (38.4)		449,568 (36.6)	429,427 (34.9)	
Ever	178,828 (14.3)	424,151 (17.9)		178,772 (14.5)	169,596 (13.8)	
Unknown	624,524 (49.8)	1,031,229 (43.6)		600,947 (48.9)	630,264 (51.3)	

aSDs were used to evaluate the balance of baseline characteristics between the two groups, with a threshold of 0.1 considered acceptable balance.

Abbreviations: aSD, absolute standardized difference; COPD, chronic obstructive pulmonary disease; SD, standard deviation.

Supplementary Table 4. Baseline characteristics of the study population by the history of atopic dermatitis

Variables	Before propensity score matching		aSD	After propensity score matching		aSD
	With atopic dermatitis (n=256,996)	Without atopic dermatitis (n=2,364,170)		With atopic dermatitis (n=256,886)	Without atopic dermatitis (n=256,886)	
Mean age (SD), y	27.8 (21.5)	42.3 (20.3)	0.69	27.8 (21.5)	28.0 (21.4)	0.01
Sex, n (%)			0.08			0.02
Female	114,508 (44.6)	1,141,860 (48.3)		114,467 (44.6)	112,275 (43.7)	
Male	142,488 (55.4)	1,222,310 (51.7)		142,419 (55.4)	144,611 (56.3)	
Region of residence, n (%)			0.01			0.00
Urban	112,587 (43.8)	1,049,501 (44.4)		112,524 (43.8)	113,053 (44.0)	
Rural	144,409 (56.2)	1,314,669 (55.6)		144,362 (56.2)	143,833 (56.0)	
Income level, n (%)			0.02			0.00
Low (≤20%)	47,737 (18.6)	457,354 (19.4)		47,703 (18.6)	47,644 (18.6)	
Middle or High (>20%)	209,259 (81.4)	1,906,816 (80.7)		209,183 (81.4)	209,242 (81.5)	
Charlson Comorbidity Index, n (%)			0.17			0.04
0	217,369 (84.6)	1,919,934 (81.2)		217,292 (84.6)	215,562 (83.9)	
1-2	20,584 (8.0)	169,115 (7.2)		20,566 (8.0)	21,316 (8.3)	
>2	19,043 (7.4)	275,121 (11.6)		19,028 (7.4)	20,008 (7.8)	
Comorbidities						
Hypertension	21,385 (8.3)	367,147 (15.5)	0.22	21,380 (8.3)	21,752 (8.5)	0.01
Dyslipidemia	25,047 (9.8)	351,653 (14.9)	0.16	25,030 (9.7)	25,998 (10.1)	0.01
Diabetes mellitus	11,812 (4.6)	195,286 (8.3)	0.15	11,808 (4.6)	12,194 (4.8)	0.01
Chronic kidney disease	956 (0.4)	13,644 (0.6)	0.03	954 (0.4)	930 (0.4)	0.00
COPD	4,133 (1.6)	30,419 (1.3)	0.03	4,122 (1.6)	4,510 (1.8)	0.01
Rheumatic disease	1,041 (0.4)	11,026 (0.5)	0.01	1,039 (0.4)	1,119 (0.4)	0.00
Co-medications						
Immunomodulator	3,355 (1.3)	10,892 (0.5)	0.09	3,258 (1.3)	3,497 (1.4)	0.01
Smoking			0.42			0.13
Never	67,947 (26.4)	908,790 (38.4)		67,934 (26.5)	65,334 (25.4)	
Ever	24,954 (9.7)	424,151 (17.9)		24,950 (9.7)	23,774 (9.3)	
Unknown	164,095 (63.9)	1,031,229 (43.6)		164,002 (63.8)	167,778 (65.3)	

aSDs were used to evaluate the balance of baseline characteristics between the two groups, with a threshold of 0.1 considered acceptable balance.

Abbreviations: aSD, absolute standardized difference; COPD, chronic obstructive pulmonary disease; SD, standard deviation.

Supplementary Table 5. Baseline characteristics of the study population by the history of food allergy

Variables	Before propensity score matching		aSD	After propensity score matching		aSD
	With food allergy (n=7,144)	Without food allergy (n=2,364,170)		With food allergy (n=7,144)	Without food allergy (n=7,144)	
Mean age (SD), y	40.5 (25.0)	42.3 (20.3)	0.08	40.5 (25.0)	41.5 (25.4)	0.04
Sex, n (%)			0.09			0.08
Female	3,124 (43.7)	1,141,860 (48.3)		3,124 (43.7)	3,206 (44.9)	
Male	4,020 (56.3)	1,222,310 (51.7)		4,020 (56.3)	3,938 (55.1)	
Region of residence, n (%)			0.15			0.03
Urban	2,637 (36.9)	1,049,501 (44.4)		2,637 (36.9)	2,729 (38.2)	
Rural	4,507 (63.1)	1,314,669 (55.6)		4,507 (63.1)	4,415 (61.8)	
Income level, n (%)			0.01			0.05
Low (≤20%)	1,416 (19.8)	457,354 (19.4)		1,416 (19.8)	1,275 (17.9)	
Middle or High (>20%)	5,728 (80.2)	1,906,816 (80.7)		5,728 (80.2)	5,869 (82.2)	
Charlson Comorbidity Index, n (%)			0.15			0.05
0	5,393 (75.5)	1,919,934 (81.2)		5,393 (75.5)	5,232 (73.2)	
1-2	733 (10.3)	169,115 (7.2)		733 (10.3)	755 (10.6)	
>2	1,018 (14.3)	275,121 (11.6)		1,018 (14.3)	1,157 (16.2)	
Comorbidities						
Hypertension	1,389 (19.4)	367,147 (15.5)	0.10	1,389 (19.4)	1,506 (21.1)	0.04
Dyslipidemia	1,434 (20.1)	351,653 (14.9)	0.14	1,434 (20.1)	1,452 (20.3)	0.01
Diabetes mellitus	794 (11.1)	195,286 (8.3)	0.10	794 (11.1)	912 (12.8)	0.05
Chronic kidney disease	47 (0.7)	13,644 (0.6)	0.01	47 (0.7)	137 (1.9)	0.10
COPD	170 (2.4)	30,419 (1.3)	0.08	170 (2.4)	167 (2.3)	0.00
Rheumatic disease	61 (0.9)	11,026 (0.5)	0.05	61 (0.9)	57 (0.8)	0.01
Co-medications						
Immunomodulator	60 (0.8)	10,892 (0.5)	0.05	60 (0.8)	96 (1.3)	0.05
Smoking			0.13			0.08
Never	2,917 (40.8)	908,790 (38.4)		2,917 (40.8)	2,644 (37.0)	
Ever	937 (13.1)	424,151 (17.9)		937 (13.1)	930 (13.0)	
Unknown	3,290 (46.1)	1,031,229 (43.6)		3,290 (46.1)	3,570 (50.0)	

aSDs were used to evaluate the balance of baseline characteristics between the two groups, with a threshold of 0.1 considered acceptable balance.

Abbreviations: aSD, absolute standardized difference; COPD, chronic obstructive pulmonary disease; SD, standard deviation.

Supplementary Table 6. Baseline characteristics of the study population by the history of chronic rhinosinusitis

Variables	Before propensity score matching		aSD	After propensity score matching		aSD
	With chronic rhinosinusitis (n=486,209)	Without chronic rhinosinusitis (n=2,364,170)		With chronic rhinosinusitis (n=486,001)	Without chronic rhinosinusitis (n=486,001)	
Mean age (SD), y	35.2 (20.4)	42.3 (20.3)	0.35	35.2 (20.4)	35.3 (20.4)	0.00
Sex, n (%)			0.06			0.00
Female	221,318 (45.5)	1,141,860 (48.3)		221,292 (45.5)	220,212 (45.3)	
Male	264,891 (54.5)	1,222,310 (51.7)		264,709 (54.5)	265,789 (54.7)	
Region of residence, n (%)			0.04			0.00
Urban	226,005 (46.5)	1,049,501 (44.4)		225,886 (46.5)	225,686 (46.4)	
Rural	260,204 (53.5)	1,314,669 (55.6)		260,115 (53.5)	260,315 (53.6)	
Income level, n (%)			0.02			0.00
Low (≤20%)	89,523 (18.4)	457,354 (19.4)		89,487 (18.4)	89,535 (18.4)	
Middle or High (>20%)	396,686 (81.6)	1,906,816 (80.7)		396,514 (81.6)	396,466 (81.6)	
Charlson Comorbidity Index, n (%)			0.14			0.03
0	391,462 (80.5)	1,919,934 (81.2)		391,462 (80.6)	390,979 (80.5)	
1-2	48,721 (10.0)	169,115 (7.2)		48,522 (10.0)	47,921 (9.9)	
>2	46,026 (9.5)	275,121 (11.6)		46,017 (9.5)	47,101 (9.7)	
Comorbidities						
Hypertension	55,126 (11.3)	367,147 (15.5)	0.12	55,126 (11.3)	55,440 (11.4)	0.00
Dyslipidemia	66,336 (13.6)	351,653 (14.9)	0.04	66,327 (13.7)	67,580 (13.9)	0.01
Diabetes mellitus	29,375 (6.0)	195,286 (8.3)	0.09	29,372 (6.0)	29,156 (6.0)	0.00
Chronic kidney disease	1,980 (0.4)	13,644 (0.6)	0.02	1,980 (0.4)	1,677 (0.4)	0.01
COPD	11,826 (2.4)	30,419 (1.3)	0.08	11,618 (2.4)	11,904 (2.5)	0.00
Rheumatic disease	2,504 (0.5)	11,026 (0.5)	0.01	2,504 (0.5)	2,124 (0.4)	0.01
Co-medications						
Immunomodulator	2,427 (0.5)	10,892 (0.5)	0.01	2,426 (0.5)	2,060 (0.4)	0.01
Smoking			0.11			0.06
Never	177,465 (36.5)	908,790 (38.4)		177,460 (36.5)	166,645 (34.3)	
Ever	71,843 (14.8)	424,151 (17.9)		71,843 (14.8)	67,583 (13.9)	
Unknown	236,901 (48.7)	1,031,229 (43.6)		236,698 (48.7)	251,773 (51.8)	

aSDs were used to evaluate the balance of baseline characteristics between the two groups, with a threshold of 0.1 considered acceptable balance.

Abbreviations: aSD, absolute standardized difference; COPD, chronic obstructive pulmonary disease; SD, standard deviation.

Supplementary Table 7. Subgroup analyses of the risk of claims-based incident asthma following COVID-19 infection after propensity score matching, by pre-existing systemic atopy and upper-airway disease

Groups	No. of patients	No. of events	Sum of PYs	IR per 1,000 PYs (95% CI)	HR (95% CI)
**Atopy/UAD burden (No. of pre-existing conditions)**					
With 1 condition					
Matched control group	1,255,839	1,789	867,751	2.06 (1.97–2.16)	1.00 (Reference)
Atopy/UAD group	1,255,839	2,814	862,393	3.26 (3.15–3.39)	1.58 (1.49–1.68)
With ≥2 conditions					
Matched control group	353,576	428	247,237	1.73 (1.58–1.90)	1.00 (Reference)
Atopy/UAD group	353,576	1,033	243,601	4.24 (3.99–4.51)	2.44 (2.18–2.73)
P-for-interaction					<0.0001
**Pre-index systemic corticosteroid dose**					
≤ Subgroup-specific mean dose					
Matched control group	1,015,978	1,428	703,117	2.03 (1.93–2.14)	1.00 (Reference)
Atopy/UAD group	1,015,978	2,168	699,238	3.10 (2.97–3.23)	1.53 (1.43–1.63)
> Subgroup-specific mean dose					
Matched control group	570,473	855	394,392	2.17 (2.03–2.32)	1.00 (Reference)
Atopy/UAD group	570,473	1,665	390,500	4.26 (4.06–4.47)	1.96 (1.81–2.13)
P-for-interaction					<0.0001
**Age**					
≤ mean age					
Matched control group	835,001	1,029	584,382	1.76 (1.66–1.87)	1.00 (Reference)
Atopy/UAD group	835,001	1,783	581,032	3.07 (2.93–3.21)	1.74 (1.61–1.88)
> mean age					
Matched control group	682,579	1,221	463,340	2.64 (2.49–2.79)	1.00 (Reference)
Atopy/UAD group	682,579	2,009	459,708	4.37 (4.18–4.57)	1.65 (1.54–1.78)
P-for-interaction					0.327
**Sex**					
Female					

Matched control group	711,503	1,261	483,704	2.61 (2.47–2.76)	1.00 (Reference)
Atopy/UAD group	711,503	2,074	479,937	4.32 (4.14–4.51)	1.66 (1.54–1.78)
Male					
Matched control group	799,257	978	559,236	1.75 (1.64–1.86)	1.00 (Reference)
Atopy/UAD group	799,257	1,722	555,852	3.10 (2.96–3.25)	1.77 (1.64–1.91)
P-for-interaction					0.232
**COVID-19 severity (ICU admission)**					
Yes (ICU admission)					
Matched control group	16,733	40	19,163	2.09 (1.53–2.85)	1.00 (Reference)
Atopy/UAD group	16,733	82	18,509	4.43 (3.57–5.50)	2.11 (1.45–3.08)
No					
Matched control group	1,546,694	2,244	1,058,640	2.12 (2.03–2.21)	1.00 (Reference)
Atopy/UAD group	1,546,694	3,725	1,055,019	3.53 (3.42–3.65)	1.67 (1.58–1.76)
P-for-interaction					0.228
**Prior COVID-19 vaccination**					
Yes					
Matched control group	1,251,931	2,010	844,851	2.38 (2.28–2.49)	1.00 (Reference)
Atopy/UAD group	1,251,931	3,401	840,717	4.05 (3.91–4.18)	1.69 (1.61–1.79)
No					
Matched control group	240,969	202	182,426	1.11 (0.97–1.27)	1.00 (Reference)
Atopy/UAD group	240,969	356	181,920	1.96 (1.76–2.17)	1.77 (1.49–2.10)
P-for-interaction					0.614

Abbreviations: Atopy/UAD, pre-existing systemic atopy and/or upper-airway disease; CI, confidence interval; COVID-19, coronavirus disease 2019; HR, hazard ratio; ICU, intensive care unit; IR, incidence rate; PY, person-years.

IRs were calculated using Poisson regression models, and HRs were estimated using Cox proportional hazards models within each stratum-specific matched cohort. P-for-interaction values were calculated using two-sided Wald tests for group-by-stratum interaction terms; no adjustment was made for multiple comparisons. Exact P values are provided where possible; P values <0.0001 are reported as <0.0001.

Supplementary Table 8. Sensitivity analyses of the risk of claims-based incident asthma following COVID-19 infection after propensity score matching, by pre-existing systemic atopy and upper-airway disease

Groups	No. of patients	No. of events	Sum of PYs	IR per 1,000 PYs (95% CI)	PS-matched HR (95% CI)
**Main analysis**					
Control group	1,563,148	2,300	1,079,289	2.13 (2.05–2.22)	1.00 (Reference)
Atopy/UAD group	1,563,148	3,808	1,073,329	3.55 (3.44–3.66)	1.66 (1.58–1.75)
**Restricted to individuals with smoking data**					
Control group	810,745	1,387	554,060	2.50 (2.38–2.64)	1.00 (Reference)
Atopy/UAD group	810,745	2,381	550,104	4.33 (4.16–4.51)	1.73 (1.61–1.84)
Never smokers					
Control group	579,258	995	395,791	2.51 (2.36–2.68)	1.00 (Reference)
Atopy/UAD group	579,258	1,712	392,975	4.36 (4.16–4.57)	1.73 (1.60–1.87)
Ever smokers					
Control group	231,507	370	158,166	2.34 (2.11–2.59)	1.00 (Reference)
Atopy/UAD group	231,507	669	157,134	4.26 (3.95–4.59)	1.82 (1.60–2.06)
**Stricter outcome definition (≥ 2 asthma diagnoses with asthma-related medication)**					
Control group	1,563,148	728	1,079,289	0.67 (0.63–0.73)	1.00 (Reference)
Atopy/UAD group	1,563,148	1,188	1,073,329	1.11 (1.05–1.17)	1.64 (1.49–1.80)
**Minimum follow-up duration of 6 months**					
Control group	1,172,281	1,943	928,842	2.09 (2.00–2.19)	1.00 (Reference)
Atopy/UAD group	1,172,281	3,116	925,228	3.37 (3.25–3.49)	1.61 (1.52–1.70)

IRs were calculated using Poisson regression models, and HRs were estimated using Cox proportional hazards models.

Abbreviations: Atopy/UAD, systemic atopy and/or upper-airway disease; CI, confidence interval; COVID-19, coronavirus disease 2019; HR, hazard ratio; IR, incidence rate; PS, propensity score; PY, person-years.

Supplementary Table 9. Sensitivity analyses of the risk of claims-based incident asthma following COVID-19 infection after propensity score matching, by pre-existing systemic atopy and upper-airway disease: Calendar-time stratified analyses (before vs after March 2022)

Groups	No. of patients	No. of events	Sum of PYs	IR per 1,000 PYs (95% CI)	PS-matched HR (95% CI)
**Main analysis**					
Control group	1,563,148	2,300	1,079,289	2.13 (2.05–2.22)	1.00 (Reference)
Atopy/UAD group	1,563,148	3,808	1,073,329	3.55 (3.44–3.66)	1.66 (1.58–1.75)
**Calendar-time stratified analyses before and after March 2022**					
Before March 2022					
Control group	391,818	768	347,824	2.21 (2.06-2.37)	1.00 (Reference)
Atopy/UAD group	391,818	1,159	344,069	3.37 (3.18-3.57)	1.52 (1.39-1.67)
After March 2022					
Control group	1,171,848	1,544	729,607	2.12 (2.01-2.22)	1.00 (Reference)
Atopy/UAD group	1,171,848	2,649	729,288	3.63 (3.50-3.77)	1.72 (1.61-1.83)

IRs were calculated using Poisson regression models, and HRs were estimated using Cox proportional hazards models.

Abbreviations: Atopy/UAD, systemic atopy and/or upper-airway disease; CI, confidence interval; COVID-19, coronavirus disease 2019; HR, hazard ratio; IR, incidence rate; PS, propensity score; PY, person-years.

Supplementary Table 10. Sensitivity analyses of the risk of claims-based incident asthma following COVID-19 infection after propensity score matching, by pre-existing systemic atopy and upper-airway disease: Lag-time analyses excluding early outcomes

Groups	No. of patients	No. of events	Sum of PYs	IR per 1,000 PYs (95% CI)	PS-matched HR (95% CI)
**Main analysis**					
Control group	1,563,148	2,300	1,079,289	2.13 (2.05–2.22)	1.00 (Reference)
Atopy/UAD group	1,563,148	3,808	1,073,329	3.55 (3.44–3.66)	1.66 (1.58–1.75)
**Lag-time analyses excluding outcomes within 3 and 6 months**					
3-month lag-time					
Control group	1,560,902	1,140	1,078,392	1.06 (1.00-1.12)	1.00 (Reference)
Atopy/UAD group	1,560,902	1,813	1,072,918	1.69 (1.61-1.77)	1.61 (1.49-1.73)
6-month lag-time					
Control group	1,559,997	548	1,078,047	0.51 (0.47-0.55)	1.00 (Reference)
Atopy/UAD group	1,559,997	907	1,072,606	0.85 (0.79-0.90)	1.69 (1.52-1.87)

IRs were calculated using Poisson regression models, and HRs were estimated using Cox proportional hazards models.

Abbreviations: Atopy/UAD, systemic atopy and/or upper-airway disease; CI, confidence interval; COVID-19, coronavirus disease 2019; HR, hazard ratio; IR, incidence rate; PS, propensity score; PY, person-years.

Supplementary Table 11. Sensitivity analyses of the risk of claims-based incident asthma following COVID-19 infection after propensity score matching, by pre-existing systemic atopy and upper-airway disease: Analyses applying 2- and 3-year washout periods

Groups	No. of patients	No. of events	Sum of PYs	IR per 1,000 PYs (95% CI)	PS-matched HR (95% CI)
**Main analysis**					
Control group	1,563,148	2,300	1,079,289	2.13 (2.05–2.22)	1.00 (Reference)
Atopy/UAD group	1,563,148	3,808	1,073,329	3.55 (3.44–3.66)	1.66 (1.58–1.75)
**Washout-period analyses (2 and 3 years)**					
2-year washout					
Control group	1,561,457	2,324	1,074,729	2.16 (2.08-2.25)	1.00 (Reference)
Atopy/UAD group	1,561,457	3,796	1,069,925	3.55 (3.44-3.66)	1.64 (1.56-1.73)
3-year washout					
Control group	1,530,395	2,193	1,031,604	2.13 (2.04-2.22)	1.00 (Reference)
Atopy/UAD group	1,530,395	3,643	1,030,927	3.53 (3.42-3.65)	1.66 (1.58-1.75)

IRs were calculated using Poisson regression models, and HRs were estimated using Cox proportional hazards models.

Abbreviations: Atopy/UAD, systemic atopy and/or upper-airway disease; CI, confidence interval; COVID-19, coronavirus disease 2019; HR, hazard ratio; IR, incidence rate; PS, propensity score; PY, person-years.

Supplementary Table 12. Sensitivity analyses of the risk of claims-based incident asthma following COVID-19 infection after propensity score matching, by pre-existing systemic atopy and upper-airway disease: Analyses with propensity score re-estimation incorporating healthcare utilization

Groups	No. of patients	No. of events	Sum of PYs	IR per 1,000 PYs (95% CI)	PS-matched HR (95% CI)
**Main analysis**					
Control group	1,563,148	2,300	1,079,289	2.13 (2.05–2.22)	1.00 (Reference)
Atopy/UAD group	1,563,148	3,808	1,073,329	3.55 (3.44–3.66)	1.66 (1.58–1.75)
**PS re-estimation with healthcare utilization***					
Control group	1,324,907	2,175	904,039	2.41 (2.31-2.51)	1.00 (Reference)
Atopy/UAD group	1,324,907	3,000	910,174	3.30 (3.18-3.42)	1.37 (1.30-1.45)

*Healthcare utilization variables included all-cause and respiratory-related outpatient visits and hospitalizations.

IRs were calculated using Poisson regression models, and HRs were estimated using Cox proportional hazards models.

Abbreviations: Atopy/UAD, systemic atopy and/or upper-airway disease; CI, confidence interval; COVID-19, coronavirus disease 2019; HR, hazard ratio; IR, incidence rate; PS, propensity score; PY, person-years.
