# Supplementary material for: Systemic atopy and upper-airway disease define susceptibility to incident asthma after COVID-19 in Korea
Source: Nat Commun. 2026 Jun 27;17:8042. doi: 10.1038/s41467-026-74860-w (PMC13454191; doi:10.1038/s41467-026-74860-w)
Supplement: Supplementary file 2 [file 41467_2026_74860_MOESM2_ESM.pdf]

nature portfolio

Peer Review File

Systemic atopy and upper-airway disease define susceptibility to incident asthma after COVID-19

Corresponding Author: Dr Young-Chan Kim

This file contains all reviewer reports in order by version, followed by all author rebuttals in order by version.

Version 0:

Reviewer comments:

Reviewer #1

(Remarks to the Author)
This is a large-scale and potentially impactful population-based analysis leveraging a nationwide Korean database. The study addresses an important question regarding post–COVID-19 respiratory sequelae. However, several major issues should be addressed to strengthen the validity of the conclusions and clarify the conceptual advance.

1. Ascertainment of SARS-CoV-2 infection
A major concern relates to potential exposure misclassification. Individuals with SARS-CoV-2 infection confirmed solely by home-based antigen testing may not have been captured in the database, particularly during later phases of the pandemic. The extent to which such under-ascertainment could bias the observed associations should be discussed, and sensitivity analyses addressing this issue would strengthen confidence in the findings.

2. Definition and validity of incident asthma
The operational definition of incident asthma requires further clarification and justification. Asthma is ideally diagnosed using objective lung function testing, including demonstration of variable airflow limitation. Reliance on diagnostic codes and/or post-COVID medication prescriptions may capture heterogeneous conditions, including transient post-infectious airway disease, bronchial hyperresponsiveness, or asthma-like symptoms, rather than true incident asthma. The authors should more clearly justify their outcome definition and discuss the potential for outcome misclassification and its impact on interpretation.

3. Conceptual advance and novelty
Respiratory infections are well recognized as triggers for subsequent asthma development. The manuscript would benefit from a clearer articulation of the novel contribution of this study beyond established knowledge. Specifically, the authors should clarify how stratification by pre-existing allergic and upper-airway phenotypes advances mechanistic understanding or alters clinical or public health perspectives on post-COVID asthma risk.

Reviewer #2

(Remarks to the Author)
Thank you for the opportunity to review this study. This manuscript is well-written and methodologically sound. Only a few points need to be clarified. Below, I have outlined my comments.

1. The authors acknowledge that their look-back period for excluding pre-existing asthma only extends to 2019. I think there may be potential misclassification of prevalent asthma. May need some sensitivity analyses (e.g., restricting to older adults who haven't sought care for years).
2. Detection bias could exist. Individuals in the Atopy/UAD group already have established patterns of healthcare utilization for respiratory issues (e.g., seeing specialists for allergic rhinitis or chronic rhinosinusitis). These patients may be more likely to receive a formal asthma diagnosis for post-COVID cough or wheeze compared to the control group, who may not seek specialized care as readily.
3. The study relies entirely on ICD-10 claims and prescription records and may not distinguish between different asthma endotypes (T2-high vs. non-T2). Furthermore, post-COVID respiratory symptoms can mimic asthma without meeting the physiological criteria of reversible airway obstruction.

4.The HR for food allergy is the highest, but the sample size is relatively small. The CI is wide. This should be emphasized as "hypothesis-generating" rather than a definitive finding compared to the rhinitis data.
5.The authors suggest higher pre-index steroid use correlates with higher risk. It would be helpful to clarify if this is a marker of disease severity (confounding by indication) or if they suspect a direct pharmacological effect on viral clearance.

Version 1:

Reviewer comments:

Reviewer #1

(Remarks to the Author)
The authors have revised the manuscript and it is acceptable for publication.

Reviewer #2

(Remarks to the Author)
Thank you for the opportunity to review this revised manuscript. The authors have addressed all my comments. I have no additional comments.

Open Access This Peer Review File is licensed under a Creative Commons Attribution 4.0 International License, which permits use, sharing, adaptation, distribution and reproduction in any medium or format, as long as you give appropriate credit to the original author(s) and the source, provide a link to the Creative Commons license, and indicate if changes were made.
In cases where reviewers are anonymous, credit should be given to 'Anonymous Referee' and the source.
The images or other third party material in this Peer Review File are included in the article’s Creative Commons license, unless indicated otherwise in a credit line to the material. If material is not included in the article’s Creative Commons license and your intended use is not permitted by statutory regulation or exceeds the permitted use, you will need to obtain permission directly from the copyright holder.
To view a copy of this license, visit https://creativecommons.org/licenses/by/4.0/

REVIEWER COMMENTS

Reviewer #1 (Remarks to the Author):

This is a large-scale and potentially impactful population-based analysis leveraging a nationwide Korean database. The study addresses an important question regarding post–COVID-19 respiratory sequelae. However, several major issues should be addressed to strengthen the validity of the conclusions and clarify the conceptual advance.

Response: The authors appreciate the Editors and Reviewers for the positive feedback on our manuscript. We are grateful for your insightful comments, and we have taken them into consideration in our revision. In the following pages, we present a point-by-point response (blue) to each comment (black), through which we hope to address your queries and suggestions.

R1-1. Ascertainment of SARS-CoV-2 infection

A major concern relates to potential misclassification of exposure. Individuals with SARS-CoV-2 infection confirmed solely by home-based antigen testing may not have been captured in the database, particularly during later phases of the pandemic. The extent to which such under-ascertainment could bias the observed associations should be discussed, and sensitivity analyses addressing this issue would strengthen confidence in the findings.

Response: We thank the reviewer for this important and thoughtful comment regarding potential misclassification of exposure. As described in the Methods section, SARS-CoV-2 infection was identified through confirmed cases recorded in the national registry. During the study period, confirmation was primarily based on laboratory polymerase chain reaction (PCR) testing, although testing policies in South Korea evolved over time; from mid-March 2022 positive rapid antigen results were accepted as confirmed cases in certain settings (Shin et al., *J Korean Med Sci* 2022; Lee et al., *Infect Chemother* 2024). Therefore, individuals whose infection was identified solely through home-based antigen testing without formal confirmation would not have been captured in our cohort.

This concern became more relevant during later phases of the pandemic, particularly during the Omicron wave, when rapid antigen testing became more widely used. We therefore selected March 2022 as a pragmatic analytical cutoff to distinguish a period in which PCR-based ascertainment remained predominant from a subsequent period in which under-ascertainment of infections identified only by antigen testing was more likely.

To address this concern, we performed calendar-time–stratified analyses comparing infections confirmed before and after 1 March 2022. The magnitude of association remained similar across the two periods and was consistent with the main findings (Supplementary Table 9). Specifically, the propensity score–matched hazard ratio was 1.52 (95% CI, 1.39–1.67) for infections confirmed before March 2022 and 1.72 (95% CI, 1.61–1.83) thereafter. Although the direction of any potential bias cannot be determined with certainty, these findings make it less likely that changes in testing practice alone account for the observed association.

In response to this comment, we revised the Methods section to clarify the infection ascertainment strategy, added calendar-time–stratified sensitivity analyses to the Results, and expanded the Discussion to acknowledge that under-ascertainment of infections detected only by home antigen testing may limit generalizability beyond confirmed cases recorded in the national registry.

**Methods (Page 17, Lines 360)**	
**Revised**	Fourth, to address potential under-ascertainment of SARS-CoV-2 infection due to home-based antigen testing without subsequent PCR confirmation, we performed calendar-time–stratified analyses comparing infections confirmed before and after 1 March 2022. This cutoff was chosen to reflect the transition period during the Omicron wave in Korea, when rapid antigen testing became more widely used and positive rapid antigen results were accepted as confirmed cases in certain settings [30, 31].

**Results (Page 5, Lines 106)**	
**Revised**	In calendar-time–stratified analyses comparing infections confirmed before and after 1 March 2022, the association remained consistent with the main findings (Supplementary Table 9). The propensity score–matched hazard ratio was 1.52 (95% CI, 1.39–1.67) before March 2022 and 1.72 (95% CI, 1.61–1.83) thereafter.

**Discussion (Page 10, Lines 207)**	
**Added**	Second, SARS-CoV-2 infection was identified through confirmed cases recorded in the national registry. During the study period, confirmation was primarily based on laboratory PCR testing, although national testing policies evolved over time; from mid-March 2022, positive rapid antigen results were also accepted as

confirmed cases in certain settings. Individuals tested solely by home-based antigen kits without formal confirmation would not have been captured. The association remained consistent across calendar periods, suggesting that testing changes are unlikely to explain the findings, although under-ascertainment of milder infections may limit generalizability.

R1-2. Definition and validity of incident asthma

The operational definition of incident asthma requires further clarification and justification. Asthma is ideally diagnosed using objective lung function testing, including demonstration of variable airflow limitation. Reliance on diagnostic codes and/or post-COVID medication prescriptions may capture heterogeneous conditions, including transient post-infectious airway disease, bronchial hyperresponsiveness, or asthma-like symptoms, rather than true incident asthma. The authors should more clearly justify their outcome definition and discuss the potential for outcome misclassification and its impact on interpretation.

Response: Thank you for the helpful comment. We agree with the reviewer that claims-based definitions cannot confirm asthma using objective lung function testing and may capture a spectrum of post-infectious airway conditions. For this reason, we have revised the manuscript to state more explicitly that our outcome reflects claims-based, clinically recognized incident asthma in routine care, rather than physiologically confirmed new-onset asthma.

Our primary outcome definition required both an ICD-10 diagnosis of asthma (J45/J46) and prescription of asthma-related medication. This operational definition has been commonly used in prior Korean NHIS-based administrative data studies (Oh et al., *Nat Commun* 2024; Kim et al., *J Allergy Clin Immunol Pract* 2024) to identify clinically recognized asthma in routine care. At the same time, we fully acknowledge that claims data cannot distinguish asthma from all other post-viral airway syndromes with complete certainty.

To further reduce potential misclassification, we conducted lag-time sensitivity analyses excluding outcomes occurring within 3 and 6 months after the index date, thereby reducing the possibility that transient post-infectious respiratory symptoms or short-term bronchial hyperresponsiveness were classified as incident asthma **(Supplementary Table 10)**. The associations remained generally consistent across these analyses (e.g., HR 1.61 [95% CI 1.49–1.73] with a 3-month lag and HR 1.69 [95% CI 1.52–1.87] with a 6-month lag), supporting the robustness of the findings. In addition, we had previously conducted an analysis using a stricter outcome definition requiring at least two asthma diagnoses with medication prescriptions, which yielded similar results (HR 1.64, 95% CI 1.49–1.80; **Supplementary**

Table 8).

Taken together, these analyses suggest that the observed association is unlikely to be explained solely by transient post-viral airway symptoms or outcome misclassification. Nevertheless, we have revised the manuscript to describe the outcome more precisely as claims-based incident asthma and to further discuss the potential for outcome misclassification in the Discussion section.

**Methods (Page 17, Lines 366)**	
**Revised**	Fifth, to reduce the possibility that transient post-infectious respiratory symptoms or short-term bronchial hyperresponsiveness were misclassified as incident asthma, we conducted lag-time sensitivity analyses excluding outcomes occurring within 3 and 6 months after the index date. In these analyses, individuals were followed from the end of each lag period, and events occurring during the lag period were excluded (Supplementary Table 10).

**Results (Page 6, Lines 110)**	
**Revised**	In lag-time sensitivity analyses starting follow-up at 3 and 6 months after the index date, the associations remained generally consistent with the primary findings (Supplementary Table 10), making it less likely that the observed association is explained solely by transient early post-infectious respiratory symptoms.

**Discussion (Page 10, Lines 203)**	
**Revised**	First, the study relied on claims-based definitions without spirometry or biomarkers and therefore cannot distinguish between asthma endotypes (e.g., T2-high versus non-T2) or confirm variable airflow limitation objectively. Our central contribution is not endotype assignment but population-level susceptibility stratification.

R1-3. Conceptual advance and novelty

Respiratory infections are well recognized as triggers for subsequent asthma development. The manuscript would benefit from a clearer articulation of the novel contribution of this study

beyond established knowledge. Specifically, the authors should clarify how stratification by pre-existing allergic and upper-airway phenotypes advances mechanistic understanding or alters clinical or public health perspectives on post-COVID asthma risk.

Response: We thank the reviewer for this important comment. Our study was motivated not simply by the established observation that respiratory infections may precipitate asthma, but by the hypothesis that pre-existing allergic and upper-airway inflammatory disease may create a susceptible airway milieu in which SARS-CoV-2 infection is more likely to be followed by clinically recognized incident asthma. In this context, the conceptual advance of our study lies in showing, at national scale, that post-COVID asthma risk is not uniform among infected survivors but is stratified by readily identifiable host phenotypes and increases in a graded manner with allergic multimorbidity burden. These findings extend the current literature by suggesting that post-COVID asthma risk may be shaped not only by infection itself but also by pre-existing host susceptibility.

Because our study includes only individuals with SARS-CoV-2 infection and uses claims-based outcome definitions, these findings should be interpreted as evidence of phenotypic risk stratification rather than direct mechanistic proof. Nonetheless, they are consistent with a biologically plausible host-susceptibility framework in which pre-existing allergic and upper-airway disease may amplify vulnerability to post-viral lower-airway disease. This phenotypic stratification also has potential clinical and public health relevance, as it may support more targeted post-COVID surveillance and earlier diagnostic evaluation in higher-risk subgroups.

In response to the reviewer's suggestion, we have revised the Introduction section and expanded the Discussion section to more explicitly articulate this conceptual advance and its implications.

**Introduction (Page 3, Lines 51–56)**	
**Before**	Although multiple cohort studies have reported an increased incidence of post-COVID-19 asthma, explicit tests of effect modification by these factors remain rare.
**After**	Although multiple cohort studies have reported an increased incidence of post-COVID-19 asthma, most have focused on overall risk estimates. Whether susceptibility to incident asthma after SARS-CoV-2 infection is heterogeneous among survivors and patterned by pre-existing allergic or upper-airway

	inflammatory phenotypes has not been systematically evaluated. Distinguishing between these possibilities is important for understanding post-COVID asthma risk and for informing targeted post-COVID surveillance.

**Discussion (Page 7, Lines 131–137)**	
**Added**	Collectively, these results indicate that susceptibility to claims-based incident asthma after SARS-CoV-2 infection is not uniformly distributed but instead is patterned by pre-existing systemic atopy and upper-airway inflammatory phenotypes. Rather than viewing post-COVID asthma solely as a direct consequence of viral injury, our findings are consistent with a host-susceptibility framework in which underlying allergic and upper-airway disease may amplify vulnerability to post-viral lower-airway disease and thereby help identify higher-risk subgroups for targeted post-COVID surveillance.

Reviewer #2 (Remarks to the Author):

Thank you for the opportunity to review this study. This manuscript is well-written and methodologically sound. Only a few points need to be clarified. Below, I have outlined my comments.

R2-1. The authors acknowledge that their look-back period for excluding pre-existing asthma only extends to 2019. I think there may be potential misclassification of prevalent asthma. May need some sensitivity analyses (e.g., restricting to older adults who haven't sought care for years).

Response: We thank the reviewer for this important methodological point. We agree that because claims history in the K-COV-N database is available from January 2019 onwards, individuals with asthma diagnosed before 2019 who generated no asthma-related claims during the available look-back window could, in principle, be misclassified as incident cases.

In this study, all participants were required to have no asthma-related claims from January 2019 until the index date, using all available claims history prior to infection. In addition, when defining prior asthma for exclusion, we applied a broad criterion of ≥1 asthma diagnosis code, whereas the study outcome required both an asthma diagnosis and a prescription for asthma-related medication. This approach was intended to maximize sensitivity in excluding individuals with potential pre-existing asthma while maintaining higher specificity in defining incident asthma cases.

To address this concern more directly, we performed an additional sensitivity analysis applying extended washout periods of 2 and 3 years before the index date (Supplementary Table 11). The results were consistent with the main findings, with hazard ratios of 1.64 (95% CI, 1.56–1.73) for the 2-year washout and 1.66 (95% CI, 1.58–1.75) for the 3-year washout. Notably, the number of eligible participants and effect estimates changed only minimally across washout definitions, further supporting the robustness of the findings.

The reviewer also suggested restricting analyses to older adults who had not sought care for several years. While we appreciate this suggestion, our primary aim was to evaluate the risk of new-onset asthma following COVID-19 infection across the general infected population. We considered that restricting the analysis to older individuals could narrow the study population and reduce generalizability. We therefore considered that extending the washout period and applying a broad exclusion definition for prior asthma provided a more direct strategy to address potential misclassification while preserving the population-based

nature of the cohort.

Taken together, these analyses suggest that potential misclassification of prevalent asthma is unlikely to influence the observed association.

**Methods (Page 17, Lines 371)**	
**Revised**	Sixth, to minimize potential misclassification of prevalent asthma due to the limited availability of claims history before 2019, we performed sensitivity analyses applying extended washout periods of 2 and 3 years before the index date (Supplementary Table 11).

**Results (Page 6, Lines 114)**	
**Revised**	In sensitivity analyses applying extended washout periods of 2 and 3 years before the index date, the association remained consistent with the primary findings (Supplementary Table 11), with hazard ratios of 1.64 (95% CI, 1.56–1.73) and 1.66 (95% CI, 1.58–1.75), respectively.

**Discussion (Page 10, Lines 223)**	
**Revised**	Fourth, because claims history was available from 2019 onward, some individuals with historically prevalent but clinically inactive asthma may have been misclassified as incident cases.

R2-2. Detection bias could exist. Individuals in the Atopy/UAD group already have established patterns of healthcare utilization for respiratory issues (e.g., seeing specialists for allergic rhinitis or chronic rhinosinusitis). These patients may be more likely to receive a formal asthma diagnosis for post-COVID cough or wheeze compared to the control group, who may not seek specialized care as readily.

Response: We agree that this is an important concern. Individuals with pre-existing Atopy/UAD may have greater baseline contact with the healthcare system, which could increase opportunities for asthma diagnosis during follow-up, independent of any true increase in disease risk.

To address potential detection bias more directly, we quantified pre-index healthcare utilization during the one year before the index date using four indicators: (1) all-cause

outpatient visits (continuous variable), (2) all-cause hospitalizations (categorical variable: 0 *vs.* ≥1), (3) respiratory-related outpatient visits (continuous variable), and (4) respiratory-related hospitalizations (categorical variable: 0 *vs.* ≥1). Respiratory-related utilization was defined using ICD-10 codes J00–J99, excluding J45–J46. These measures were selected to capture both general intensity of healthcare contact and respiratory-specific opportunities for diagnostic evaluation.

We then re-estimated propensity scores with additional inclusion of these utilization variables and repeated 1:1 nearest-neighbor matching. The association was attenuated but remained elevated (Supplementary Table 12; HR 1.37, 95% CI 1.30–1.45), suggesting that differences in baseline healthcare contact do not fully explain the observed association.

**Methods (Page 17, Lines 374)**	
**Revised**	Finally, to evaluate the potential for detection bias arising from differences in baseline healthcare utilization, we re-estimated propensity scores with additional inclusion of healthcare utilization measures during the one year prior to the index date and repeated 1:1 nearest-neighbor matching. These measures included all-cause outpatient visits (continuous), all-cause hospitalizations (categorical, 0 *vs.* ≥1), respiratory-related outpatient visits (continuous), and respiratory-related hospitalizations (categorical, 0 *vs.* ≥1). Respiratory-related healthcare utilization was defined using ICD-10 codes J00–J99, excluding J45–J46 (Supplementary Table 12).

**Results (Page 6, Lines 117)**	
**Revised**	After propensity score re-matching incorporating pre-index healthcare utilization, the association was attenuated but remained elevated (HR 1.37, 95% CI 1.30–1.45; Supplementary Table 12).

R2-3. The study relies entirely on ICD-10 claims and prescription records and may not distinguish between different asthma endotypes (T2-high vs. non-T2). Furthermore, post-COVID respiratory symptoms can mimic asthma without meeting the physiological criteria of reversible airway obstruction.

Response: Thank you for the reviewer's helpful comment. We agree that claims data do not include spirometry or biomarker data and cannot distinguish asthma endotypes (for example, T2-high versus non-T2) or confirm variable airflow limitation objectively. We have revised the Discussion to state more explicitly that our outcome represents claims-based, clinically recognized asthma recorded in routine care and that any endotype-level or mechanistic inference remains speculative. Importantly, our central contribution is not endotype assignment but population-level susceptibility stratification, showing that readily identifiable allergic and upper-airway phenotypes help identify a higher-risk subgroup for claims-based incident asthma after SARS-CoV-2 infection.

We also agree that some post-COVID respiratory symptoms may overlap with asthma-like presentations without fulfilling physiological criteria for asthma. This concern is precisely why we performed several sensitivity analyses designed to reduce the likelihood that transient or nonspecific post-infectious respiratory symptoms were counted as incident asthma: (1) lag-time sensitivity analyses excluding outcomes occurring within 3 and 6 months after the index date (Supplementary Table 10), in which the associations remained generally consistent (e.g., HR 1.61 [95% CI 1.49–1.73] with a 3-month lag and HR 1.69 [95% CI 1.52–1.87] with a 6-month lag), and (2) a stricter outcome definition requiring ≥2 asthma diagnoses with medication prescription (Supplementary Table 8; HR 1.64, 95% CI 1.49–1.80). Across these approaches, the associations remained consistent with the primary analysis in both direction and magnitude, reducing concern that the findings are driven by transient post-viral airway symptoms alone.

We now emphasize more clearly that future studies integrating spirometry, biomarkers, and detailed clinical phenotyping will be necessary to clarify the underlying endotypes and mechanisms.

**Discussion (Page 10, Lines 198–203)**	
**Before**	First, the study relied on claims-based definitions without spirometry or biomarkers.
**After**	First, the study relied on claims-based definitions without spirometry or biomarkers and therefore cannot distinguish between asthma endotypes (e.g., T2-high versus non-T2) or confirm variable airflow limitation objectively. Our central contribution is not endotype assignment but population-level susceptibility stratification. Future studies integrating spirometry, biomarkers, and detailed

	clinical phenotyping will be necessary to clarify the underlying endotypes and mechanisms.

R2-4. The HR for food allergy is the highest, but the sample size is relatively small. The CI is wide. This should be emphasized as "hypothesis-generating" rather than a definitive finding compared to the rhinitis data.

Response: We thank the reviewer for this important comment. We agree that although the hazard ratio (HR) for food allergy was numerically the highest among the evaluated conditions, the relatively small sample size and limited number of events resulted in a wide confidence interval, reflecting statistical imprecision.

We have revised the Discussion section to more clearly acknowledge this limitation. Specifically, we now emphasize that the association observed for food allergy should be interpreted cautiously and considered hypothesis-generating rather than definitive, particularly in comparison to the more precisely estimated associations for allergic rhinitis and chronic rhinosinusitis.

**Discussion (Page 8, Lines 155–159)**	
**Before**	The higher risks observed with atopic dermatitis (HR 1.84) and food allergy (HR 2.81) are consistent with an adult analogue of the "atopic march," wherein systemic atopy predisposes individuals to later airway disease.
**After**	The higher risk observed with atopic dermatitis is consistent with an adult analogue of the "atopic march." Although the estimate for food allergy was less precise because of the small number of events, its direction was broadly aligned with the same systemic atopy framework. However, given the wide confidence interval, the food allergy finding should be interpreted cautiously as hypothesis-generating and requires confirmation in larger cohorts.

R2-5. The authors suggest higher pre-index steroid use correlates with higher risk. It would be helpful to clarify if this is a marker of disease severity (confounding by indication) or if they suspect a direct pharmacological effect on viral clearance.

Response: We appreciate the reviewer's insightful comment. Our primary interpretation is that

higher pre-index systemic corticosteroid exposure reflects underlying disease activity/severity (confounding by indication), rather than a direct causal pharmacologic effect on viral clearance. To improve clarity, we have revised the Discussion to emphasize the "severity marker" interpretation and to avoid causal wording.

Importantly, pre-index systemic corticosteroid exposure was defined using indication-specific prescriptions—systemic corticosteroids dispensed in claims carrying the corresponding Atopy/UAD diagnosis codes—to minimize capture of corticosteroids prescribed for unrelated conditions. This strengthens its interpretation as a proxy for activity/severity of the underlying allergic/upper-airway disease. While prior studies have suggested that systemic corticosteroids may be associated with delayed viral clearance, our observational data cannot disentangle pharmacologic effects from disease severity and therefore do not support causal inference.

**Discussion (Page 9, Lines 175–186)**	
**Before**	The stronger association among those with higher pre-index systemic corticosteroid exposure is most consistent with confounding by indication, as individuals with more active or severe allergic disease/chronic rhinosinusitis are both more likely to receive systemic corticosteroids and to be at greater post-viral risk. However, a direct pharmacologic contribution cannot be excluded, since systemic glucocorticoids have been linked to delayed SARS-CoV-2 viral clearance in some studies, potentially prolonging epithelial injury.
**After**	The stronger association among those with higher pre-index systemic corticosteroid exposure most likely reflects confounding by indication: individuals with more active or severe allergic disease are both more likely to receive systemic corticosteroids and more likely to have greater underlying disease activity that predisposes to post-viral asthma. Importantly, pre-index systemic corticosteroid exposure was defined using indication-specific prescriptions—systemic corticosteroids dispensed in claims carrying the corresponding Atopy/UAD diagnosis codes—to minimize capture of corticosteroids prescribed for unrelated conditions. This supports its interpretation as a proxy for disease activity and severity. While prior studies have suggested that systemic corticosteroids may be associated with delayed viral clearance, our observational data cannot disentangle pharmacologic effects from disease severity and therefore do not support causal inference regarding a direct pharmacologic

pathway.
